# Complete Blood Count-Derived Inflammation Indexes Are Useful in Predicting Metabolic Syndrome in Children and Adolescents with Severe Obesity

**DOI:** 10.3390/jcm13072120

**Published:** 2024-04-05

**Authors:** Alice Marra, Adele Bondesan, Diana Caroli, Alessandro Sartorio

**Affiliations:** 1 Experimental Laboratory for Auxo-Endocrinological Research, Istituto Auxologico Italiano, IRCCS (Istituto di Ricovero e Cura a Carattere Scientifico), 28824 Verbania, Italy; a.bondesan@auxologico.it (A.B.); d.caroli@auxologico.it (D.C.); sartorio@auxologico.it (A.S.); 2 Experimental Laboratory for Auxo-Endocrinological Research, Istituto Auxologico Italiano, IRCCS (Istituto di Ricovero e Cura a Carattere Scientifico), 20145 Milan, Italy

**Keywords:** pediatric obesity, metabolic syndrome, biomarkers, children, adolescents, blood cell count, high-density lipoprotein, cardiovascular risk

## Abstract

**Background:** Childhood obesity is a globally increasing pathological condition leading to long-term health issues such as cardiovascular diseases and metabolic syndrome (MetS). This study aimed to determine the clinical value of the Complete Blood Count-derived inflammation indexes Monocyte/HDL-C ratio (MHR), Lymphocyte/HDL-C ratio (LHR), Neutrophil/HDL-C ratio (NHR), and System Inflammation Response Index (SIRI) to predict the presence of metabolic syndrome and its association with cardiovascular risk markers (HOMA-IR, TG/HDL-C, and non-HDL-C) in children and adolescents with obesity. **Methods:** The study included a total of 552 children/adolescents with severe obesity (BMI: 36.4 [32.7–40.7] kg/m^2^; 219 males, 333 females; age: 14.8 [12.9−16.3] years), who were further subdivided based on the presence or absence of metabolic syndrome (MetS+ and MetS respectively). **Results:** The MHR, LHR, and NHR indexes (*p* < 0.0001), but not SIRI (*p* = 0.524), were significantly higher in the MetS+ compared to the MetS− subgroup, showing a positive correlation with the degree of MetS severity (*p* < 0.0001). Furthermore, MHR, LHR, and NHR were positively associated with cardiometabolic risk biomarkers (HOMA-IR: MHR *p* = 0.000, LHR *p* = 0.001, NHR *p* < 0.0001; TG/HDL-C: MHR, LHR, NHR *p* < 0.000; non-HDL-C: MHR, LHR *p* < 0.0001, NHR *p* = 0.000). Finally, the ROC curve analysis demonstrated that among the analyzed indexes, only MHR, LHR, and NHR had diagnostic value in distinguishing MetS patients among children and adolescents with obesity (MHR: AUC = 0.7045; LHR: AUC = 0.7205; NHR: AUC = 0.6934; *p* < 0.0001). **Conclusions:** In conclusion, the MHR, LHR, and NHR indexes, but not the SIRI index, can be considered useful tools for pediatricians to assess the risk of MetS and cardiometabolic diseases in children and adolescents with obesity and to develop multidisciplinary intervention strategies to counteract the widespread disease.

## 1. Introduction

Childhood obesity has emerged as a significant concern in developed countries, reaching epidemic proportions, with over 340 million children diagnosed as obese in 2020 [1].

Importantly, pediatric obesity significantly increases the long-term risk of developing metabolic disorders, including insulin resistance, dyslipidemia, hypertension, and glucose intolerance, collectively known as metabolic syndrome (MetS) [2,3,4]. MetS is a serious multifaced condition that is on the rise among children and adolescents, mirroring the increasing prevalence of obesity [4,5,6].

Although the precise onset of the disease is still unknown, it is suggested that the buildup of free fatty acids in different tissues contributes to compromised insulin signaling and consequent insulin resistance [2,7]. Additionally, studies indicate that inflammatory cytokines released from malfunctioning adipocytes, like monocyte chemoattractant protein-1 and tumor necrosis factor-alpha, stimulate macrophage migration to adipose tissues, amplifying cytokine production. Additionally, the decrease in adiponectin levels associated with obesity can worsen inflammatory responses in adipose tissues [2,7].

The combination of these factors collectively amplifies the risk of cardiovascular disease (CVD) in adulthood [8,9,10].

Recent research has shifted focus towards identifying early indicators of MetS and cardiovascular risk in children with obesity. Of particular interest are inflammatory markers derived from the complete blood count (CBC), which provide valuable insights into the systemic inflammation associated with obesity [11,12,13,14,15,16].

Indexes like the Monocyte/HDL-C ratio (MHR), Lymphocyte/HDL-C ratio (LHR), Neutrophil/HDL-C ratio (NHR), and System Inflammation Response Index (SIRI) have emerged as relevant biomarkers for assessing systemic inflammation and forecasting cardiovascular risk in a cost-effective manner [12,14,15,17,18,19,20,21].

While most of the research utilizing these indexes has concentrated on adults [12,14,15,17,18,19,20,21,22], to date there is a paucity of studies examining the correlation between childhood obesity and CBC-derived inflammation indexes in relation to MetS and cardiometabolic risks.

Therefore, the present study aimed to assess the utility of CBC-derived inflammatory indexes in stratifying and predicting outcomes, exploring their intricate relationships with childhood obesity, MetS, and cardiovascular risk factors.

## 2. Material and Methods

### 2.1. Patients

In this retrospective study, a cohort of 552 children and adolescents diagnosed with severe obesity (219 males and 333 females) was examined. The median age (interquartile range) of the participants was 14.8 years (12.9–16.3), with a median BMI (body mass index) (interquartile range) of 36.4 (32.7–40.7). These individuals were admitted to the Division of Auxology, IRCCS Istituto Auxologico Italiano, Piancavallo-Verbania, Italy, for a 3-week multidisciplinary integrated body weight reduction program (BWRP). The program involved an integrated approach, including an energy-restricted diet, moderate aerobic exercise as part of physical rehabilitation, psychological counseling, and nutritional education [23,24].

Obesity was defined as the presence of a BMI ≥ 97th percentile for gender and chronological age by using the Italian growth charts [25]. Exclusion criteria encompassed conditions such as renal and hepatic failure, secondary obesity (e.g., genetic obesity), acute infections, inflammatory, autoimmune, or malignant diseases, neurodegenerative disorders, as well as hematological and/or oncological conditions.

The diagnosis of MetS was based on the IDF (International Diabetes Federation) criteria for pediatric populations [26,27].

For individuals aged < 16 years:

1. abdominal obesity (waist circumference ≥ 90th percentile);
2. triglycerides: ≥150 mg/dL (1.7 mmol/L) or specific treatment for lipid abnormalities;
3. HDL-C (High Density Lipoprotein): <40 mg/dL (1.03 mmol/L);
4. blood pressure: SBP (systolic blood pressure) ≥ 130 mmHg or DBP (diastolic blood pressure) ≥ 85 mmHg and/or treatment for previously diagnosed hypertension;
5. fasting plasma glucose (FPG) concentration ≥ 100 mg/dL (5.6 mmol/L) or previously diagnosed type 2 diabetes mellitus.

For individuals aged ≥ 16 years:

1. abdominal obesity (waist circumference ≥ 94 cm for males; ≥80 cm for females);
2. triglycerides: ≥150 mg/dL (1.7 mmol/L) or specific treatment for this lipid abnormality;
3. HDL-C: <40 mg/dL (1.03 mmol/L) for males and <50 mg/dL (<1.29 mmol/L) for females, or specific treatment for lipid abnormalities;
4. blood pressure: SBP (systolic blood pressure) ≥ 130 mmHg or DBP (diastolic blood pressure) ≥ 85 mmHg and/or treatment for previously diagnosed hypertension;
5. fasting plasma glucose (FPG) concentration ≥ 100 mg/dL (5.6 mmol/L) or previously diagnosed type 2 diabetes mellitus.

Patients were divided into three subgroups based on the number of MetS criteria: MetS 0–2 (0–2 MetS criteria), MetS 3 (3 MetS criteria), and MetS 4–5 (4–5 criteria). Patients falling into the MetS 0–2 subgroup were classified as MetS negative (MetS−), while those categorized in the MetS 3 and MetS 4–5 subgroups were considered MetS-positive (MetS+).

This retrospective study was approved by the Ethical Committee of Istituto Auxologico Italiano, Milan, Italy (research code: 2022_03_15_05, acronym: REEOBEPED). All data included in the present analysis were collected from the medical records of patients hospitalized at our institution in the past. Informed assent (children/adolescents) and consent (parents) were obtained at the time of hospitalization for the use of clinical and laboratory data on the patient.

### 2.2. Anthropometric Measurements

Waist circumference (WC), height, and body weight were determined following the guidelines outlined in the Anthropometric Standardization Reference Manual [28]. Standing height was measured using a stadiometer-equipped scale (Holtain Limited, Crymych, Dyfed, UK). Body weight was recorded to the nearest 0.1 kg utilizing an electronic scale (Ro WU 150, Wunder Sa.bi., Trezzo sull’Adda, Italy). Waist circumference was measured in a standing position with a non-elastic flexible tape measure, with participants gently exhaling midway between the lowest rib and the top of the iliac crest [29].

### 2.3. Laboratory Analyses and Metabolic Variables

Blood samples were collected using standard tubes after an overnight fast, followed by the determination of blood count and metabolic variables. Hematologic parameters were assessed using Beckman Coulter instruments. Leukocyte counts were conducted using the impedance-based method after erythrocyte (RBC) lysis, while leukocyte populations were identified based on volume, conductivity, and scatter properties using VCS technology.

Serum HDL-C and triglyceride levels were determined through colorimetric enzymatic assays from Roche Diagnostics, Monza, Italy. Serum insulin concentration was measured via a chemiluminescent immunometric assay employing a commercial kit (Elecsys Insulin, Roche Diagnostics, Monza, Italy), while serum glucose levels were determined using the glucose oxidase enzymatic method (Roche Diagnostics, Monza, Italy). All serum analyses for triglycerides, HDL-C, and insulin were conducted using the Roche Cobas 6000 analyzer.

The calculation of NHR, MHR, LHR, and SIRI involved the following formulas:

- Neutrophil/HDL-C ratio (NHR) = neutrophil count (10^9^/L)/HDL-C (mg/dL);
- Monocyte/HDL-C ratio (MHR) = monocyte count (10^9^/L)/HDL-C (mg/dL);
- Lymphocyte/HDL-C ratio (LHR) = lymphocyte count (10^9^/L)/HDL-C (mg/dL);
- Systemic Inflammation Response Index (SIRI) = monocyte (10^9^/L) × neutrophil (10^9^/L)/lymphocyte count (10^9^/L).

Cardiovascular risk indexes were calculated as follows:

- Homeostatic model assessment of insulin resistance (HOMA-IR) = fasting insulin (mU/L) × fasting glucose (mmol/L)/22.5;
- TG/HDL-C ratio = total triglycerides (mg/dL)/HDL-C (mg/dL);
- non-HDL-C = total cholesterol (mg/dL) − HDL-C (mg/dL).

### 2.4. Blood Pressure Measurement

Systolic (SBP) and diastolic (DBP) blood pressure were measured three times while the subject was seated and relaxed by using a sphygmomanometer equipped with a cuff of appropriate size placed on the right arm [30].

### 2.5. Statistical Analysis

**Figure 1:**
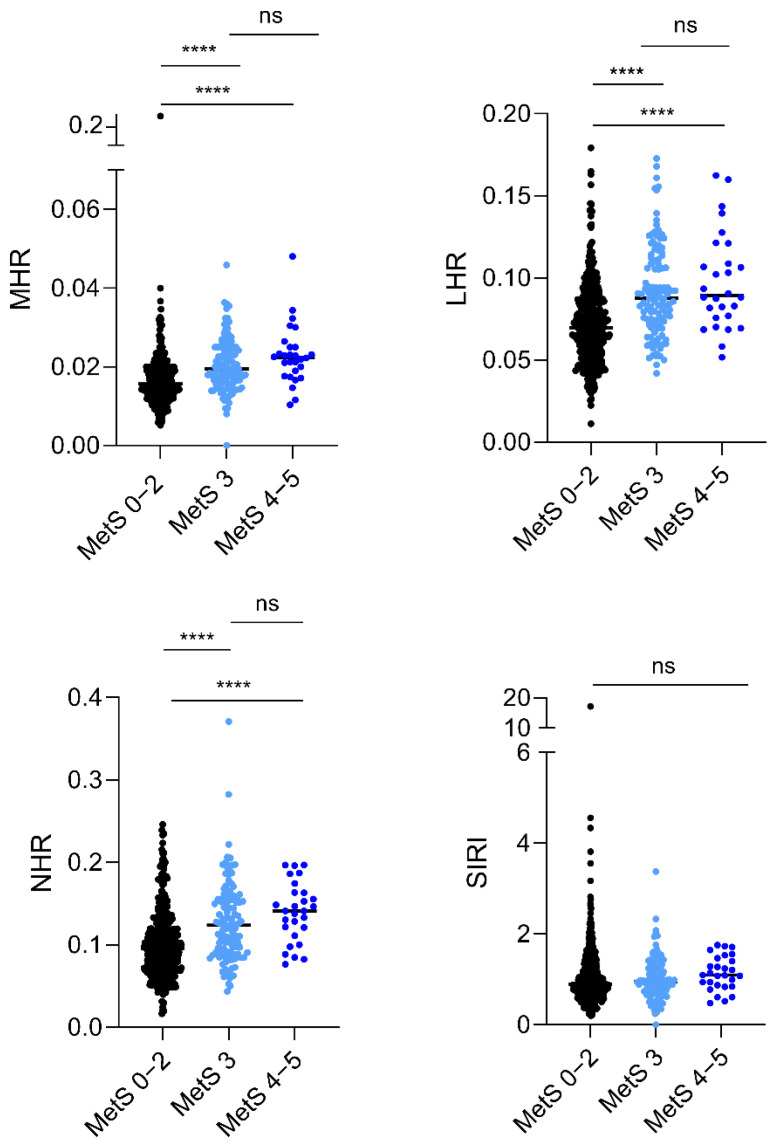
Relationships between the degrees of MetS and the CBC-derived inflammation indexes in children and adolescents with severe obesity. Dot plots display median values of these indexes corresponding to MetS severity levels: MetS 0–2 (low grade, absence of MetS, in black), MetS 3 (minimal MetS, in light blue), and MetS 4–5 (full MetS, in dark blue). The difference between the MetS subgroups for each index was determined using the non-parametric Mann–Whitney U test. *p* ≤ 0.001; **** = *p* ≤ 0.0001; ns (not significant) = *p* > 0.05. Abbreviations: MetS: metabolic syndrome; MetS 0–2: patients with 0 to 2 altered criteria for MetS; MetS 3: patients with 3 altered criteria for MetS; MetS 4–5: patients with 4 to 5 altered criteria for MetS; MHR: monocytes-to-HDL-C ratio; LHR: lymphocytes-to-HDL-C ratio; NHR: neutrophils-to-HDL-C ratio; SIRI: systemic immune response index; r: correlation coefficient.

**Figure 2:**
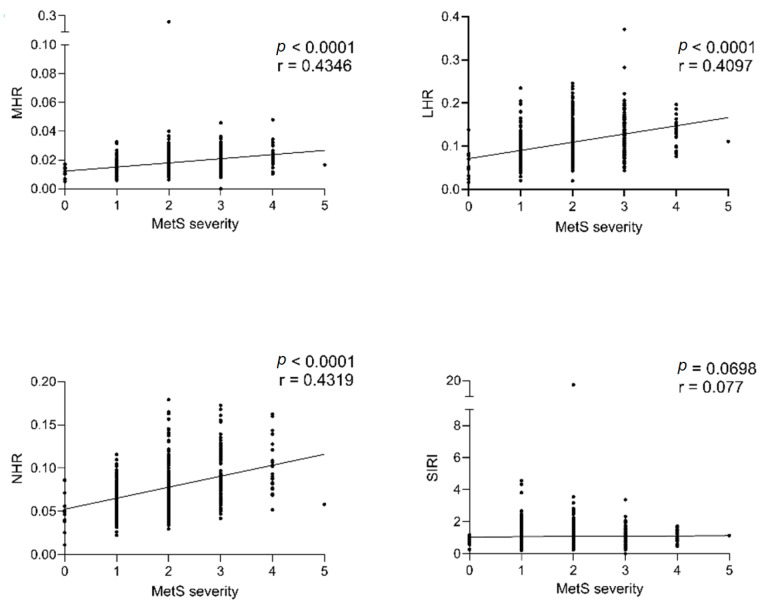
Correlation between CBC-derived inflammation indexes and degrees of MetS severity. The non-parametric Spearman’s rank correlation test demonstrated a positive correlation between CBC-derived inflammation indexes and the degrees of MetS severity. Significance was defined as *p* ≤ 0.05 for all analyses with a 95% confidence interval. Abbreviations: MHR: monocytes-to-HDL-C ratio; LHR: lymphocytes-to-HDL-C ratio; NHR: neutrophils-to-HDL-C ratio; SIRI: systemic immune response index; r: correlation coefficient; *p*: *p*-value.

**Figure 3:**
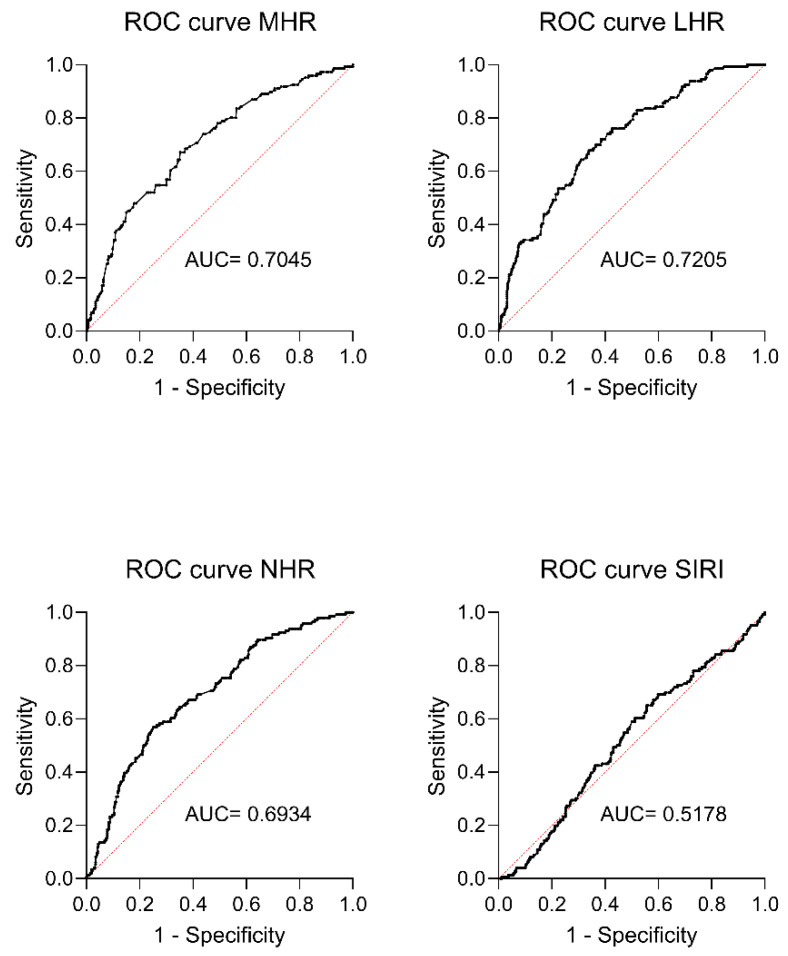
ROC curve analysis of CBC-derived inflammation indexes to predict metabolic syndrome (MetS) in severely obese adolescents. The analysis revealed significant predictive abilities for identifying MetS using MHR, LHR, and NHR (MHR *p* < 0.0001; LHR *p* < 0.0001; NHR *p* < 0.0001), but not with SIRI (*p* = 0.524). A *p*-value (*p*) ≤ 0.05 was considered significant. Abbreviations: ROC: receiver operating characteristic analysis; AUC: area under the curve; MHR: monocytes-to-HDL-C ratio; LHR: lymphocytes-to-HDL-C ratio; NHR: neutrophils-to-HDL-C ratio; SIRI: systemic immune response index.

GraphPad Prism 10.2.0 software for Windows (GraphPad Software, San Diego, CA, USA, https://www.graphpad.com/, (accessed on 8 August 2023)) was utilized for data analysis and visualization. The normal distribution and linearity of each variable were assessed using the Shapiro–Wilk normality test. As normality assumptions were not met, descriptive statistics for continuous and categorical variables in Table 1, Table 2, Table 3 and Table 4, Tables S1 and S2, and Figure 1, Figure 2 and Figure 3 were presented as medians (interquartile range) or percentages.

Continuous variables were evaluated and compared among subgroups (obese MetS-/obese MetS+, degrees of MetS severity) using the non-parametric Mann–Whitney U test, while sex was considered a categorical variable. A two-way ANOVA was employed to explore the impact of sex on MetS prevalence. Fisher’s exact test was utilized for comparing contingency tables and categorical variables.

The non-parametric Spearman’s rank correlation test was employed to assess the correlation between CBC-derived inflammation indexes and the degrees of MetS severity, as well as the cardiometabolic risk factors. Binary logistic regression analysis was performed to determine the predictive value of CBC-derived inflammation indexes (considered independent variables) for MetS.

Receiver operating characteristic (ROC) curves were constructed to illustrate the ability to discriminate between MetS− and MetS+. Graphs depicting the raw data of CBC-derived inflammation indexes of MetS− and MetS+ were generated, and the area under the ROC curves (AUC) was calculated to evaluate the accuracy of these indexes. Optimal cut-off values for CBC-derived inflammation indexes, along with AUC values, sensitivity, and specificity for predicting MetS, were determined using the same data.

A significance level of two-tailed *p*-value ≤ 0.05 was adopted for all data analyses, with a 95% confidence interval applied to all datasets. Before patient selection, a power analysis was conducted. A sample size of at least 50 subjects for each group (MetS+ or MetS−) was determined to be adequate to detect a statistically significant mean difference of 0.004 for MHR, with a standard deviation of 0.07 (for both groups), using a *t*-test with a power of 80% and an α error of 0.05.

## 3. Results

This retrospective study included a total of 552 subjects with severe obesity (no. children: 27/552; 14 males, 13 females; median (interquartile range): 8.7 [7.4–9.4] years); no. adolescents: 525/552; 205 males, 320 females; median (interquartile range): 15.0 [13.1–16.4]). A total of 146 subjects (68 males, 78 females) were diagnosed with MetS (MetS+), while 406 (151 males, 255 females) were not affected (MetS−). The clinical and biochemical data of the study population are shown in Table 1.

**Table 1:** Demographic, biochemical, and clinical characteristics of the study group (all obese, obese MetS−, obese MetS+).

Parameters	All Obese (No. 552)	Obese MetS− (No. 406, 73.6%)	Obese MetS+ (No. 146, 26.4%)	*p*-Value
Age (years)	14.8 [12.9–16.3]	14.5 [12.4–15.9]	15.8 [14.0–16.9]	<0.0001
Sex (*n*, %)	M 219, 40; F 333, 60	M 151, 69; F 255, 77	M 68, 31; F 78, 23	0.523
BMI (kg/m^2^)	36.4 [32.7–40.7]	35.5 [32.2–39.8]	39.3 [35.6–42.6]	<0.0001
WC (cm)	113.0 [103.0–123.0]	110.0 [101.0–120.0]	122.0 [112.0–132.0]	<0.0001
SBP (mmHg)	120.0 [120.0–130.0]	120.0 [110.0–125.0]	130 [130.0–140.0]	<0.0001
DBP (mmHg)	80.0 [70.0–80.0]	80.0 [70.0–80.0]	80.0 [80.0–87.5]	<0.0001
TG (mg/dL)	88.0 [65.0–115.0]	80.5 [62.0–103.3]	117.0 [86.0–158.0]	<0.0001
FBG (mmol/L)	4.5 [4.3–4.3]	4.5 [4.3–4.3]	4.5 [4.3–4.8]	0.929
Insulin (mU/L)	13.1 [8.5–19.1]	11.4 [7.9–17.3]	16.4 [12.1–22.3]	<0.0001
HDL-C (mg/dL)	41.0 [35.0–48.0]	44.0 [39.8–51.0]	35.0 [32.0–38.0]	<0.0001
LDL-C (mg/dL)	99.0 [83.0–121.0]	98.0 [82.0–119.0]	103.0 [85.0–127.0]	0.063
Total cholesterol (mg/dL)	160.0 [141–181.8]	158.5 [141.0–180.3]	162.5 [139.8–185.3]	0.528

The *p*-value represents the comparison between the obese MetS− subgroup and the obese MetS+ subgroup calculated with the non-parametric Mann–Whitney U test. Fisher’s exact test was utilized to assess the impact of gender on MetS prevalence (*p* = 0.523). A *p*-value (*p*) ≤ 0.05 was considered statistically significant. Abbreviations: MetS−: children/adolescents with severe obesity without MetS; MetS+: children/adolescents with severe obesity and MetS; M: males; F: females; BMI: body mass index (kg/m^2^); WC: waist circumference (cm); SBP: systolic blood pressure (mmHg); DBP: diastolic blood pressure (mmHg); TG: triglyceride (mmol/L); FBG: fasting blood glucose (mmol/L); HDL-C: high-density lipoprotein (mmol/L); LDL-C: low-density lipoprotein.

The MetS+ subgroup had significantly higher values (median (interquartile range) of BMI (36.4 [32.7–40.7] kg/m^2^; *p* < 0.0001), waist circumference (113.0 [103.0–123.0] cm; *p* < 0.0001), blood pressure (diastolic: 80.0 [70.0–80.0] mmHg, *p* < 0.0001; systolic: 120.0 [120.0–130.0] mmHg, *p* < 0.0001), triglycerides (88.0 [65.0–115.0] mg/dl, *p* < 0.0001), and insulin (16.4 [12.1–22.3], *p* < 0.0001)) than those found in the MetS− subgroup, while the HDL-C values were significantly lower (41.0 [35.0–48.0] mg/dL, *p* < 0.0001). No significant differences were found in fasting blood glucose (*p* = 0.929), total cholesterol (*p* = 0.528), or LDL-C (*p* = 0.063) between the two subgroups.

According to the IDF’s age-specific criteria for defining MetS, the male population had a significantly higher prevalence of subjects with MetS compared to the female population (*p* = 0.046, Table 1). However, gender was not a factor in discriminating between the different distributions of MetS.

Despite the different prevalence of males and females in the population, gender was not a factor discriminating between the different distributions of MetS (*p* = 0.523).

The prevalence of MetS among the whole study population with obesity was 26.4%, with waist circumference (no. patients = 533/552, 96.6%) being the most frequently altered parameter, followed by HDL-C (no. patients = 263/552, 47.6%), blood pressure (no. patients = 232/552, 42.0%), triglycerides (no. patients = 51/552, 9.2%), and fasting blood glucose (no. patients = 5/552, 0.9%).

The total white blood cell count, CBC-derived inflammation indexes, and cardiometabolic biomarkers in the whole study population and in the subgroups with and without metabolic syndrome (MetS+ and MetS−, respectively) are shown in Table 2.

**Table 2:** Hematologic parameters, CBC-derived inflammation indexes, and cardiometabolic biomarkers of participants.

Parameters	All Obese (No. 552)	Obese MetS− (No. 406, 73.6%)	Obese MetS+ (No. 146, 26.4%)	*p*-Value
Leukocytes (10^9^/L)	8.3 [7.1–9.6]	8.2 [7.0–9.6]	8.5 [7.1–9.8]	0.217
Neutrophils (10^9^/L)	4.2 [3.4–5.2]	4.1 [3.4–5.2]	4.3 [3.4–5.3]	0.204
Lymphocytes (10^9^/L)	3.0 [2.6–3.6]	3.0 [2.6–3.5]	3.0 [2.6–3.7]	0.375
Monocytes (10^9^/L)	0.7 [0.6–0.8]	0.7 [0.6–0.8]	0.7 [0.6–0.8]	0.766
Eosinophils (10^9^/L)	0.2 [0.1–0.3]	0.2 [0.1–0.3]	0.2 [0.1–0.3]	0.664
Basophils (10^9^/L)	0.0 [0.0–0.0]	0.0 [0.0–0.0]	0.0 [0.0–0.1]	0.181
MHR	0.016 [0.013–0.20]	0.015 [0.012–0.019]	0.020 [0.017–0.025]	<0.0001
LHR	0.074 [0.058–0.091]	0.069 [0.054–0.085]	0.088 [0.073–0.111]	<0.0001
NHR	0.10 [0.07–0.13]	0.095 [0.07–0.12]	0.13 [0.09–0.15]	<0.0001
SIRI	0.92 [0.68–1.3]	0.90 [0.68–1.31]	0.98 [0.72–1.30]	0.524
HOMA-IR	2.6 [1.7–3.8]	2.3 [1.6–3.6]	3.2 [2.3–4.5]	<0.0001
non-HDL-C	117.0 [98.0–141.0]	114.0 [96.0–136.3]	126.0 [105.8–152.0]	<0.0001
TG/HDL-C	2.1 [1.4–3.0]	1.8 [1.3–2.5]	3.4 [2.4–4.7]	<0.0001

Presentation of the hematologic parameters, CBC-derived inflammation indexes, and cardiometabolic parameters. The *p*-value represents the comparison between the obese MetS− subgroup and the obese MetS+ subgroup calculated with the non-parametric Mann–Whitney U test. A *p*-value (*p*) ≤ 0.05 was considered statistically significant. Abbreviations: MetS-: children/adolescents with severe obesity without MetS; MetS+: children/adolescents with severe obesity and MetS; MHR: monocytes-to-HDL-C ratio; LHR: lymphocytes-to-HDL-C ratio; NHR: neutrophils-to-HDL-C ratio; SIRI: systemic immune response index; HOMA-IR: homeostasis model assessment-estimated insulin resistance; non-HDL-C: non-HDL cholesterol (total cholesterol—HDL-C); TG/HDL-C: triglycerides-to-HDL-C ratio.

In the MetS+ subgroup, the total white blood cell count and all the subpopulations were comparable to that of the MetS− subgroup (Table 2).

However, patients with MetS (MetS+) had significantly higher amounts of the monocytes-to-HDL-C ratio (MHR), lymphocytes-to-HDL-C ratio, and neutrophils-to-HDL-C ratio (NHR) (Table 2, *p* < 0.0001), while no difference was found for SIRI (Table 2, *p* = 0.524).

Furthermore, the cardiometabolic indexes TG/HDL ratio, HOMA-IR, and non-HDL-C ratio were significantly higher in the MetS+ subgroup than in the MetS− subgroup (*p* < 0.0001) (Table 2).

The whole population was subdivided into three subgroups based on the number of altered criteria indicating the absence (or presence) of MetS (MetS 0–2, MetS 3, and MetS 4–5).

The individuals with 0–2 altered criteria were 73.6% (no. patients = 406/552), while those with 3 altered criteria were 21.4% (no. patients = 118/552) and those with 4–5 altered criteria were 5.0% (no. patients = 28/552). The biochemical, clinical, and hematological characteristics are presented in Supplementary Tables S1 and S2.

The MHR, LHR, and NHR indexes were notably higher in patients with 3 and 4–5 altered criteria in comparison to the MetS 0–2 subgroup (Figure 1, *p* < 0.0001). No differences were found comparing the MetS 3 with the MetS 4–5 subgroup (Figure 1**,** MHR: *p* = 0.061; LHR: *p* = 0.416; NHR: *p* = 0.101). The SIRI index was comparable in the three MetS subgroups (Figure 1, MetS 0–2 vs. MetS 4–5: *p* = 0.069; MetS 0–2 vs. MetS 3: *p* = 0.939; MetS 3 vs. MetS 4–5: *p* = 0.073).

Additionally, the MHR, LHR, and NHR indexes were positively and significantly correlated with the number of MetS altered criteria (Figure 2, *p* < 0.0001).

Moreover, MHR, LHR, and NHR demonstrated a positive correlation with all the cardiometabolic markers used in the study (Table 3, HOMA-IR: MHR *p* = 0.000, LHR *p* = 0.001, NHR *p* < 0.0001; TG/HDL-C: MHR, LHR, NHR *p* < 0.0001; non-HDL-C: MHR, LHR *p* < 0.0001, NHR *p* = 0.000), while SIRI positively and significantly correlated only with the HOMA-IR index (Table 3, HOMA-IR: *p* = 0.001; TG/HDL-C: *p* = 0.512; non-HDL-C: *p* = 0.605).

**Table 3:** Correlation analysis between CBC-derived inflammation indexes and cardiometabolic risk factors in the study population.

	HOMA-IR		Non-HDL-C		TG/HDL-C	
CBC-Index	r	*p*	r	*p*	r	*p*
MHR	0.1547	0.000	0.1689	<0.0001	0.5054	<0.0001
LHR	0.1338	0.001	0.2091	<0.0001	0.5569	<0.0001
NHR	0.2393	<0.0001	0.1578	0.000	0.4479	<0.0001
SIRI	0.1393	0.001	0.02203	0.606	0.0279	0.513

The non-parametric Spearman’s test displayed the correlation between the MHR, LHR, NHR, and SIRI inflammation indexes and the HOMA-IR, TG/HDL-C, and non-HDL-C risk factors. Abbreviations: MHR: monocytes-to-HDL-C ratio; LHR: lymphocytes-to-HDL-C ratio; NHR: neutrophils-to-HDL-C ratio; SIRI: systemic immune response index; r correlation coefficient; CBC-index: Complete Blood Count-Derived Inflammation Index. The statistical difference was given with a *p*-value (*p*) ≤ 0.05. The confidence interval considered was 95%.

Binary logistic regression analysis between the CBC-derived inflammation indexes and MetS in the population with obesity revealed that MHR (*p* = 0.000), LHR (*p* < 0.0001), and NHR (*p* < 0.0001) were significantly related to MetS, but not SIRI (*p* = 0.380) (Table 4).

**Table 4:** Binary logistic regression analysis of the predicting variables for the presence of MetS.

**Exposure Variables**	OR (95% CI)	***p*** **-Value**
MHR	4.14 [4.55−3.77]	0.000
LHR	6.74 [2.67−1.70]	<0.0001
NHR	2.79 [2.93−2.67]	<0.0001
SIRI	0.88 [0.63−1.12]	0.382

Abbreviations: OR: odds ratio; CI: confidence interval; MHR: monocytes-to-HDL-C ratio; LHR: lymphocytes-to-HDL-C ratio; NHR: neutrophils-to-HDL-C ratio; SIRI: systemic immune response index; The statistical difference was given with a *p*-value (*p*) ≤ 0.05.

Finally, ROC curve analysis revealed that MHR, LHR, and NHR had a significant discriminative ability in correctly identifying MetS in children/adolescent patients (Figure 3, MHR: AUC = 0.7045 [95% CI 0.6557 to 0.7535, sensitivity 70.55%, specificity 69.95%, *p* < 0.0001]; LHR: AUC = 0.7205 [95% CI 0.6737 to 0.7674, sensitivity 74.66%, specificity 71.92%, *p* < 0.0001]; NHR: AUC = 0.6934, [95% CI 0.6447 to 0.7421, sensitivity 71.92%, specificity 69.21%, *p* < 0.0001]), while SIRI had a low diagnostic value (Figure 3, AUC = 0.5178 [95% CI 0.4642 to 0.5713, sensitivity 54.11%, specificity 51.23%, *p* = 0.524]).

## 4. Discussion

The present study sheds light on the complex interplay between inflammation and metabolic syndrome (MetS) in pediatric obesity. Our analysis of children and adolescents with severe obesity unveiled significant associations between the CBC-inflammatory biomarkers MHR, LHR, and NHR and MetS, providing valuable insights into the underlying pathophysiology of cardiometabolic complications in this population.

MetS represents a cluster of cardiovascular risk factors associated with insulin resistance, primarily influenced by factors such as visceral obesity and systemic inflammation, which often manifest early in childhood and adolescence and worsen during adulthood [2,6].

In individuals with MetS, particularly when coupled with obesity, the continuous release of cytokines and adipokines from adipose tissue triggers an inflammatory cascade involving various immune system-related cells [31,32], like neutrophils, lymphocytes, monocytes, and macrophages [33,34]. In recent years, as an alternative to the standard markers of inflammatory status (i.e., IL-6, CRP, and adiponectin), an increasing body of research has indicated that biomarkers derived from peripheral blood cells, such as the Neutrophil/Lymphocyte Ratio (NLR) [14,35], Platelet/Lymphocyte Ratio (PLR) [36], Monocyte/Lymphocyte Ratio (MLR) [21], Aggregate Systemic Inflammation Index (AISI) [17], SIRI [12,15], and Systemic Immune-Inflammation Index (SII) [12,21], can serve as indicators for detecting and predicting the presence and severity of systemic inflammatory processes, including cardiovascular diseases and MetS.

Moreover, markers associated with HDL-C were also reported to serve as reliable indicators reflecting the degree of inflammation [15,17,19,21,37,38,39,40]. Notably, alterations in lipid profiles among individuals with obesity include increases in serum total cholesterol, triglyceride, and LDL-cholesterol levels, coupled with a decrease in HDL-cholesterol levels [26,41,42].

However, evidence regarding these indicators and MetS primarily pertains to adult populations, with few conflicting findings regarding low-grade inflammation associated with pediatric MetS and its role in promoting cardiovascular disease.

For example, Turkkan et al. [35] reported that inflammatory hematological indexes, including NLR, PLR, MLR, and monocyte/HDL-cholesterol values, in adolescents with obesity were comparable to those recorded in normal-weight individuals. Mărginean et al. [11] also found comparable NLR values between children and adolescents with obesity and normal-weight individuals. In a study performed on adolescents with obesity, Dilek et al. [43] reported that PLR values were comparable between adolescents with obesity and healthy controls, while Yazaki et al. [44] detected no correlation between NLR and BMI in their study of children with obesity. In contrast, Aydin et al. [45] observed a significant increase in NLR and PLR values in adolescents with obesity compared to healthy controls.

The aforementioned disparities can be attributed to variations in study populations, including differences in sample sizes, age ranges, levels of obesity, gender distributions, and the presence or absence of MetS.

The present study aimed to assess the diagnostic value of CBC-derived inflammation indexes (MHR, LHR, NHR, and SIRI) in detecting the MetS presence in adolescents with severe obesity and their association with well-known cardiovascular risk biomarkers (HOMA-IR [46,47], non-HDL-C [48] and TG/HDL-C [49]).

The accurate assessment of MetS prevalence in children and adolescents with obesity remains challenging due to the lack of standardized criteria. This ambiguity has led to discrepancies in the reported MetS prevalence in different studies [50,51].

The prevalence of MetS among children with obesity varies widely in the literature, with reported rates ranging from 10% to 66%, influenced by the criteria used for the assessment [50,51]. Consistent with these reports, our study found a MetS prevalence of 27.9% among adolescents with severe obesity. Specifically, according to the IDF criteria, the prevalence of obesity in childhood ranged from 16.4% to 44.2%, with a higher occurrence in males compared to females (31% males vs. 23% females in this study) [51,52].

In our study, a MetS prevalence of 26.4% was found among all patients with obesity, with waist circumference being the most frequently altered parameter, affecting 96.6% of the sample (no. patients: 533/552), followed by alterations in HDL-C levels detected in 47.6% of the patients (no. of patients: 263/552). Interestingly, a relatively low prevalence of increased triglycerides was found (9.2%, no. patients: 51/552), consistent with previous findings suggesting a low prevalence of this risk parameter in adolescents with MetS [50,53]. The prevalence of elevated blood pressure (42.0%, no. of patients: 232/552) and fasting blood glucose levels (0.9%, no. of patients: 5/552) in our study closely matched those previously reported in the literature [4,12,50,54,55,56,57].

In the present study, higher values of inflammatory indexes, particularly the monocytes-to-HDL-C ratio (MHR), lymphocytes-to-HDL-C ratio (LHR), and neutrophils-to-HDL-C ratio (NHR), were detected in individuals with MetS compared to those without MetS, thus suggesting a potential role of inflammation in MetS development and progression among children/adolescents with obesity.

While it is widely recognized that obesity is frequently associated with persistent, low-grade systemic inflammation, particularly exacerbated in the presence of MetS [58], our research revealed that children/adolescents with severe obesity and MetS+ did not differ in terms of white blood cell (WBC) count or subpopulations compared to individuals with obesity without MetS.

By contrast, patients with MetS had significantly higher amounts of the monocyte-to-HDL-C ratio (MHR), lymphocyte-to-HDL-C ratio, neutrophil-to-HDL-C ratio (NHR), thus highlighting the central role of HDL-C as an indicator of altered inflammation status. Notably, these parameters appeared to be primarily influenced by reduced HDL-C levels in MetS-positive individuals rather than by increased circulating leukocytes.

Spearman correlation analysis and binary logistic regression revealed a positive association between all the indexes (with the exception of SIRI) and the presence and severity of MetS. Furthermore, ROC curve analysis corroborated these findings by demonstrating significant predictive capability for LHR, MHR, and NHR, but not for SIRI, within our MetS+ population.

Systemic inflammation plays a crucial role in the pathogenesis of chronic diseases, serving as a key factor in initiating and exacerbating their progression [59,60,61].

Currently, there is a growing interest in assessing the potential significance of hematological pro-inflammatory markers for diagnosing and predicting various chronic conditions [62,63].

To investigate this aspect further, we assessed correlations between CBC-derived inflammation indexes and cardiovascular risk biomarkers (i.e., HOMA-IR, TG/HDL-C, and non-HDL-C). Our analysis revealed significant positive correlations between LHR, NHR, and MHR with all three cardiovascular biomarkers. These findings are consistent with previous research in adult populations, indicating that systemic inflammation indexes are associated with a higher risk of cardiovascular diseases [17,19,21,38,64].

Strengths and limitations should be carefully considered in our study. We have achieved an adequate sample size through meticulous power analysis, allowing for robust conclusions drawn from analyzing a great number of children/adolescents with severe obesity (no. of patients: 552) recruited in a single third-level center for the multidisciplinary treatment of obesity. Additionally, all the parameters were measured at the same central laboratory, thus eliminating the risk of variations between different laboratory settings.

However, it is important to underline some limitations. Firstly, the lack of a control group of normal-weight individuals (which was not the aim of the present study) prevented understanding the possible BMI-related differences between individuals with obesity and those with normal weight. Furthermore, the lack of longitudinal data prevented the establishment of the chronological sequence of MetS onset and development. Additionally, the inconsistent collection of information regarding the onset and duration of MetS treatment among participants represented an aspect that will need to be investigated with targeted future research. Despite our efforts to account for various potential confounding factors and considering the young age of the patients, we cannot definitively rule out the possibility of other variables influencing MetS that may affect the concentrations of CBC-derived inflammation indexes. Given the rapid physiological changes during puberty [65,66], including alterations in secondary sexual characteristics and hormonal profiles, the evaluation of these factors could be a goal for future research.

While our ROC curve analysis suggests the predictive utility of CBC-derived inflammation indexes for children and adolescents with obesity and metabolic syndrome, it should not be regarded as the sole definitive test to establish the clinical validity of these biomarkers. Exploring additional inflammation markers, such as cytokines and oxidation parameters, would be advantageous. Unfortunately, these measurements were not available in the recruited study group, but they can be taken into consideration for new prospective studies on the population of pediatric patients with obesity. Lastly, it is noteworthy that our study focused on children/adolescents with severe obesity within the Italian context. Therefore, our findings may not be directly applicable to populations with different degrees of obesity in different countries.

In conclusion, our study offers initial evidence supporting the reliability of the MHR, LHR, and NHR indexes in predicting MetS among children and adolescents with severe obesity. Moreover, the associations observed between these indexes and cardiometabolic risk factors seem to provide valuable insights for the precise assessment of MetS linked to obesity. Altogether, the results of the present study underscore the validity of the CBC-derived inflammation indexes, which are routinely easily obtainable and at a low cost, in predicting MetS in pediatric subjects with obesity. Early identification of MetS in childhood and a deeper knowledge of the intricate relationships between childhood obesity, MetS, and cardiovascular risk factors can contribute to the development of more targeted nutritional and/or pharmacological interventions aimed at preventing the long-term cardiometabolic complications of pediatric severe obesity.

## Data Availability

The datasets used and/or analyzed during the current study will be available on the Zenodo repository, (http://www.zenodo.org) upon a reasonable request to the corresponding author.

## Supplementary Materials

The following supporting information can be downloaded at: https://www.mdpi.com/article/10.3390/jcm13072120/s1, Table S1: Demographic, biochemical, and clinical characteristics of the study subgroups (MetS 0-2, MetS 3, MetS 4-5). Table S2: Hematologic parameters, CBC-derived inflammation indexes, and cardiometabolic biomarkers of the study subgroups (MetS 0-2, MetS 3, MetS 4-5).

## Abbreviations

MetS: metabolic syndrome; CBC: complete blood count; WBC: white blood cell; BMI: body mass index; NHR: neutrophil to high-density lipoprotein; MHR: monocytes to high-density lipoprotein; LHR: lymphocytes to high-density lipoprotein; SIRI: systemic inflammation response index; HDL-C: high-density lipoprotein; HOMA-IR: homeostatic model assessment for insulin resistance; TG/HDL-C: triglyceride/high-density lipoprotein cholesterol; FPG: fasting plasma glucose; BP: blood pressure; SBP: systolic blood pressure; DBP: diastolic blood pressure; WC: waist circumference; TG: triglycerides; ROC: receiver operating characteristic; AUC: area under the curve; *p*: *p*-value; IDF: International Diabetes Federation.
