# Supplementary material for: Complete Blood Count-Derived Inflammation Indexes Are Useful in Predicting Metabolic Syndrome in Children and Adolescents with Severe Obesity
Source: J Clin Med. 2024 Apr 5;13(7):2120. doi: 10.3390/jcm13072120 (PMC11012534; doi:10.3390/jcm13072120)
Supplement: Supplementary file 1 [file jcm-13-02120-s001.zip › jcm-2936587-supplementary.pdf]

Supplementary Table S1. Demographic, biochemical, and clinical characteristics of the study subgroups (MetS 0-2, MetS 3, MetS 4-5).

Parameters	MetS 0-2 (no. 406, 73.6%)	MetS 3 (no. 118, 21.4%)	MetS 4-5 (no. 28, 5.0%)
Age (years)	14.5 [12.4–15.9]	15.8 [14.1–16-8]	15.8 [13.6–16.9]
Sex (no.)	M 151, F 255	M 57; F 61	M 11; F 17
BMI (kg/m^2)	35.5 [32.2–39.8]	39.2 [35.2–42.4]	40.0 [36.3–42.7]
WC (cm)	110.0 [101.0–120.0]	121.5 [110.0–131.3]	121.0 [115.0–133.5]
SBP (mmHg)	120.0 [110.0–125.0]	130.0 [130.0–140.0]	130.0 [130.0–140.0]
DBP (mmHg)	80.0 [70.0–80.0]	80.0 [80.0–90.0]	80.0 [80.0–80.0]
TG (mg/dL)	80.5 [62.0–103.3]	105.5 [78.2–128.3]	169.0 [157.8–195.3]
FBG (mmol/L)	4.5 [4.3–4.3]	4.5 [2.3–4.7]	4.6 [4.1–4.8]
Insulin (mU/L)	11.4 [7.9-17.3]	15.4 [11.0–21.3]	19.6 [15.6–25.1]
HDL-C (mg/dL)	44.0 [39.8–51.0]	35.0 [32.0–38.0]	34.5 [31.2–37.7]
LDL-C (mg/dL)	98.0 [82.0-119.0]	102.0 [84.7–123.0]	116.0 [97.2–137.5]
Total cholesterol (mg/dL)	158.5 [141.0–180.3]	158.0 [137.8–181.5]	179.0 [160.0–198.8]

The characteristics of the different MetS severity subgroups are displayed. The values are presented as median [interquartile range].

Abbreviations: MetS: metabolic syndrome; MetS 0–2: patients with 0 to 2 altered criteria for MetS; MetS 3: patients with 3 altered criteria for MetS; MetS 4–5: patients with 4 to 5 altered criteria for MetS; M: males; F: females; BMI: body mass index (kg/m^2); WC: waist circumference (cm); SBP: systolic blood pressure (mmHg); DBP: diastolic blood pressure (mmHg); TG: triglyceride (mg/dL); FBG: fasting blood glucose (mmol/L); HDL-C: high-density lipoprotein (mg/dL); LDL: low-density lipoprotein (mg/dL).

Supplementary Table S2. Hematologic parameters, CBC-derived inflammation indexes, and cardiometabolic biomarkers of the study subgroups (MetS 0-2, MetS 3, MetS 4-5).

Parameters	MetS 0-2 (no. 406, 73.6%)	MetS 3 (no. 118, 21.4%)	MetS 4-5 (no. 28, 5%)
Leukocytes (10^9/L)	8.2 [7.0–9.6]	8.4 [7.1–9.6]	9.0 [7.7–10.1]
Neutrophils (10^9/L)	4.1 [3.4–5.2]	4.2 [3.4–5.2]	4.8 [3.9–5.6]
Lymphocytes (10^9/L)	3.0 [2.6–3.5]	3.0 [2.6–3.7]	3.0 [2.7–4.2]
Monocytes (10^9/L)	0.7 [0.6–0.8]	0.7 [0.6–0.8]	0.8 [0.7–0.9]
Eosinophils (10^9/L)	0.2 [0.1–0.3]	0.2 [0.1–0.3]	0.2 [0.1–0.3]
Basophils (10^9/L)	0.0 [0.0–0.0]	0.0 [0.0–0.1]	0.0 [0.0–0.1]
MHR	0.015 [0.012–0.019]	0.020 [0.020 –0.020]	0.020 [0.020 –0.030]
LHR	0.069 [0.054–0.085]	0.090 [0.077–0.110]	0.090 [0.080–0.117]
NHR	0.095 [0.07–0.12]	0.120 [0.09–0.16]	0.14 [0.11–0.16]
SIRI	0.90 [0.68–1.31]	0.94 [0.69–1.29]	1.09 [0.85–1.45]
HOMA-IR	2.3 [1.6–3.6]	3.1 [2.1–4.2]	3.9 [3.0–4.9]
non-HDL-C	114.0 [96.0–136.3]	121.0 [103.8–146.3]	146.5 [122.5–167.8]
TG/HDL-C	1.8 [1.3–2.5]	3.1 [2.2–3.9]	5.3 [4.5–6.1]

Hematologic parameters, CBC-derived inflammation indexes, and cardiometabolic parameters of the MetS subgroups are displayed.
Abbreviations: MetS: metabolic syndrome; MetS 0–2: patients with 0 to 2 altered criteria for MetS; MetS 3: patients with 3 altered criteria for MetS; MetS 4–5: patients with 4 to 5 altered criteria for MetS; MHR: monocytes-to-HDL-C ratio; LHR: lymphocytes-to-HDL-C ratio; NHR: neutrophils-to-HDL-C ratio; SIRI: systemic immune response index; HOMA-IR: homeostasis model assessment-estimated insulin resistance; non-HDL-C: non-HDL cholesterol: (total cholesterol – HDL-C); TG/HDL-C: triglycerides-to-HDL-C ratio.
